# GSA STUDENT CHAPTERS: A BROAD APPROACH TO MAKING THIS NEW INITIATIVE SUCCESSFUL AT YOUR INSTITUTION—US AND GLOBALLY

**DOI:** 10.1093/geroni/igae098.1321

**Published:** 2024-12-31

**Authors:** Marilyn Gugliucci

**Affiliations:** Chair

## Abstract

The Gerontological Society of America (GSA) Board of Directors approved the formation of a committee to explore the creation of GSA Student Chapters. As of academic year 2023-24, Phase II of the GSA Student Chapter project is underway and going strong. In this symposium, four GSA student chapter advisors will be presenting on their journey as an advisor in their second year of this project, or just starting up a chapter. The topics will cover undergraduate, post-graduate, and graduate students, as well as students focused on gerontology and health professions education. Presentations include: (1) Opportunities for Integrating Sigma Phi Omega Honor Society Chapter with a GSA Student Chapter; (2) Starting a GSA Student Chapter with Post-Graduate Students in the UK: Opportunities and Challenges; (3) GSA Student Chapter as a Gateway to Undergraduate Student Understanding of Professional Organization Involvement; and (4) Health Professions and Medical Education: How the GSA Student Chapter Enhances Professionalism and Networking.

